# Supplementary material for: Explanation of observational data engenders a causal belief about smoking and cancer
Source: PeerJ. 2018 Sep 12;6:e5597. doi: 10.7717/peerj.5597 (PMC6139016; doi:10.7717/peerj.5597)
Supplement: Supplemental Information 1 [file peerj-06-5597-s001.docx]

**Title:** **Explanation of observational data engenders a causal belief about smoking and cancer**

**Supplementary Information**

Tables 2-5 show students results for a randomized quiz question asking them to identify the type of data analysis. The quiz question described an inferential analysis. Students were randomized to see or not see explanatory language that hypothesized why the association occurred.

Students were also randomized to see 3 of 4 possible incorrect answers (descriptive, causal, predictive, mechanistic) in addition to the correct answer (inferential). As a result, there were four groups of students who saw the exact same answer choices.

Results for two of these groups are presented in the accompanying paper (Tables 2 and 3 below). Tables 4 and 5 show results for the remaining two groups of students. For all groups where students were allowed to identify the analysis as causal in nature, the results of both the original and replication experiment are consistent (Tables 2, 4, and 5).
