# Supplementary material for: Explanation of observational data engenders a causal belief about smoking and cancer
Source: PeerJ. 2018 Sep 12;6:e5597. doi: 10.7717/peerj.5597 (PMC6139016; doi:10.7717/peerj.5597)
Supplement: Supplemental Information 2 [file peerj-06-5597-s002.docx]

**Results for students with answer choices of inferential, causal, predictive, and mechanistic (from accompanying paper)**

**Original experiment (January 2013):** Among students selecting from inferential, causal, predictive, and mechanistic answer choices, the majority (68.5%) correctly answered that the description referred to an inferential data analysis (Table 1). However, a significantly higher percentage of students who were shown the explanatory language claimed it was a causal analysis compared to students who did not see the additional language: 31.8% compared to 16.6% (95% CI for difference in two proportions: 12.8% - 17.5%). These results indicate that explanatory language increases the chance a student will mistake an inferential result as causal. In this case students who saw the additional explanation were almost twice as likely to claim the results as causal.

**Replication experiment (October 2013):** Again, the majority of students (70.0%) correctly answered that the description referred to an inferential data analysis (Table 1). As in the original experiment, a significantly higher percentage of students who were shown the explanatory language claimed it was a causal analysis compared to students who did not see the additional language: 34.2% compared to 14.3% (95% CI for difference in two proportions: 11.5% - 28.3%).

Table 1: Results for students with answer choices: inferential, causal, predictive, mechanistic

|  |  | January 2013 course  (N=5088) | | October 2013 course  (N=437) | |
| --- | --- | --- | --- | --- | --- |
| This is an example of a/an _________ data analysis. | | Saw explanatory language  (N=2516) | No explanatory language  (N=2572) | Saw explanatory language  (N=199) | No explanatory language  (N=238) |
|  |  |  |  |  |  |
|  | inferential | 1508 (59.9%) | 1977 (76.9%) | 116 (58.3%) | 190 (79.8%) |
|  | causal | 799 (31.8%) | 427 (16.6%) | 68 (34.2%) | 34 (14.3%) |
|  | predictive | 120 (4.8%) | 138 (5.4%) | 8 (4.0%) | 11 (4.6%) |
|  | mechanistic | 89 (3.5%) | 30 (1.2%) | 7 (3.5%) | 3 (1.3%) |
