# Supplementary material for: Explanation of observational data engenders a causal belief about smoking and cancer
Source: PeerJ. 2018 Sep 12;6:e5597. doi: 10.7717/peerj.5597 (PMC6139016; doi:10.7717/peerj.5597)
Supplement: Supplemental Information 3 [file peerj-06-5597-s003.docx]

**Results for students with answer choices of inferential, predictive, descriptive, and mechanistic (from accompanying paper)**

**Original experiment (January 2013):** Among students who weren’t given the option to select a causal as an answer (selecting instead from inferential, predictive, descriptive, and mechanistic analyses), a higher percentage (84.6%) correctly answered that the description referred to an inferential data analysis (Table 2). In this case, a significantly higher percentage of students correctly claimed the analysis was inferential when not shown the explanatory language: 88.2% compared to 80.9% (95% confidence for difference in two proportions: 5.2% - 9.3%). These results indicate that, even without the ability to identify the analysis as causal, students had a harder time correctly identifying an inferential study when given hypothesized information about the reason for a correlation. The size of the effect is must smaller than with the causal answer option, however.

**Replication experiment (October 2013):** Again, the majority of students (82.9%) correctly answered that the description referred to an inferential data analysis (Table 2). As in the original experiment, a higher percentage of students correctly claimed the analysis was inferential when not shown the explanatory language, but the results were not statistically significant: 85.3% compared to 80.4% (95% CI for difference in two proportions: -2.9% - 12.6%).

Table 2: Results for students with answer choices: inferential, predictive, descriptive, mechanistic

|  |  | January 2013 course  (N=5016) | | October 2013 course  (N=416) | |
| --- | --- | --- | --- | --- | --- |
| This is an example of a/an _________ data analysis. | | Saw explanatory language  (N=2485) | No explanatory language  (N=2531) | Saw explanatory language  (N=199) | No explanatory language  (N=217) |
|  |  |  |  |  |  |
|  | inferential | 2011 (80.9%) | 2232 (88.2%) | 160 (80.4%) | 185 (85.3%) |
|  | predictive | 196 (7.9%) | 181 (7.2%) | 10 (5.0%) | 12 (5.5%) |
|  | descriptive | 138 (5.6%) | 82 (3.2%) | 14 (7.0%) | 14 (6.5%) |
|  | mechanistic | 140 (5.6%) | 36 (1.4%) | 15 (7.5%) | 6 (2.8%) |
