# Supplementary material for: Explanation of observational data engenders a causal belief about smoking and cancer
Source: PeerJ. 2018 Sep 12;6:e5597. doi: 10.7717/peerj.5597 (PMC6139016; doi:10.7717/peerj.5597)
Supplement: Supplemental Information 4 [file peerj-06-5597-s004.docx]

**Results for students with answer choices of inferential, causal, predictive, and descriptive**

**Original experiment (January 2013):** Among students selecting from inferential, causal, predictive, and descriptive answer choices, the majority (67.9%) correctly answered that the description referred to an inferential data analysis (Table 3). However, a significantly higher percentage of students who were shown the explanatory language claimed it was a causal analysis compared to students who did not see the additional language: 30.5% compared to 16.0% (95% CI for difference in two proportions: 12.2% - 16.8%). These results indicate that explanatory language increases the chance a student will mistake an inferential result as causal. In this case students who saw the additional explanation were almost twice as likely to claim the results as causal.

**Replication experiment (October 2013):** Again, the majority of students (68.5%) correctly answered that the description referred to an inferential data analysis (Table 3). As in the original experiment, a significantly higher percentage of students who were shown the explanatory language claimed it was a causal analysis compared to students who did not see the additional language: 28.3% compared to 14.0% (95% CI for difference in two proportions: 6.4% - 22.2%).

Table 3: Results for students with answer choices: inferential, causal, predictive, descriptive

|  |  | January 2013 course  (N=5061) | | October 2013 course  (N=447) | |
| --- | --- | --- | --- | --- | --- |
| This is an example of a/an _________ data analysis. | | Saw explanatory language  (N=2581) | No explanatory language  (N=2480) | Saw explanatory language  (N=233) | No explanatory language  (N=214) |
|  |  |  |  |  |  |
|  | inferential | 1575 (61.0%) | 1862 (75.1%) | 141 (60.5%) | 165 (77.1%) |
|  | causal | 786 (30.5%) | 396 (16.0%) | 66 (28.3%) | 30 (14.0%) |
|  | predictive | 129 (5.0%) | 143 (5.8%) | 13 (5.6%) | 13 (6.1%) |
|  | descriptive | 91 (3.5%) | 79 (3.2%) | 13 (5.6%) | 6 (2.8%) |
