# Supplementary material for: Explanation of observational data engenders a causal belief about smoking and cancer
Source: PeerJ. 2018 Sep 12;6:e5597. doi: 10.7717/peerj.5597 (PMC6139016; doi:10.7717/peerj.5597)
Supplement: Supplemental Information 5 [file peerj-06-5597-s005.docx]

**Results for students with answer choices of inferential, causal, descriptive, and mechanistic**

**Original experiment (January 2013):** Among students selecting from inferential, causal, descriptive, and mechanistic answer choices, the majority (68.5%) correctly answered that the description referred to an inferential data analysis (Table 4). However, a significantly higher percentage of students who were shown the explanatory language claimed it was a causal analysis compared to students who did not see the additional language: 33.0% compared to 17.2% (95% CI for difference in two proportions: 13.4% - 18.1%). These results indicate that explanatory language increases the chance a student will mistake an inferential result as causal. In this case students who saw the additional explanation were almost twice as likely to claim the results as causal.

**Replication experiment (October 2013):** Again, the majority of students (69.3%) correctly answered that the description referred to an inferential data analysis (Table 4). As in the original experiment, a significantly higher percentage of students who were shown the explanatory language claimed it was a causal analysis compared to students who did not see the additional language: 32.6% compared to 17.8% (95% CI for difference in two proportions: 6.6% - 23.0%).

Table 4: Results for students with answer choices: inferential, causal, descriptive, mechanistic

|  |  | January 2013 course  (N=5092) | | October 2013 course  (N=463) | |
| --- | --- | --- | --- | --- | --- |
| This is an example of a/an _________ data analysis. | | Saw explanatory language  (N=2540) | No explanatory language  (N=2552) | Saw explanatory language  (N=233) | No explanatory language  (N=230) |
|  |  |  |  |  |  |
|  | inferential | 1513 (59.6%) | 1976 (77.4%) | 142 (60.9%) | 179 (77.8%) |
|  | causal | 837 (33.0%) | 439 (17.2%) | 76 (32.6%) | 41 (17.8%) |
|  | descriptive | 104 (4.1%) | 108 (4.2%) | 9 (3.9%) | 7 (3.0%) |
|  | mechanistic | 86 (3.4%) | 29 (1.1%) | 6 (2.6%) | 3 (1.3%) |
